# Acceptability of a lifelogging wearable camera in older adults with mild cognitive impairment: a mixed-method study

**DOI:** 10.1186/s12877-019-1132-0

**Published:** 2019-04-16

**Authors:** Olga Gelonch, Mireia Ribera, Núria Codern-Bové, Sílvia Ramos, Maria Quintana, Gloria Chico, Noemí Cerulla, Paula Lafarga, Petia Radeva, Maite Garolera

**Affiliations:** 1 Clinical Research Group for Brain, Cognition and Behavior, Consorci Sanitari de Terrassa, Carretera Torrebonica, S/N, 08227 Terrassa, Spain; 2 Department of Mathematics and Computer Science, University of Barcelona, Barcelona, Spain; 3 Terrasa University School of Nursing and Occupational Therapy, Autonomous University of Barcelona, Terrassa, Spain; 4 AVAN Foundation, Sabadell, Spain

**Keywords:** Mild cognitive impairment, Lifelogging, Technology, Acceptability, Mixed-method study

## Abstract

**Background:**

The main objective of this research was to evaluate the acceptance of technology based on a wearable lifelogging camera in a sample of older adults diagnosed with mild cognitive impairment (MCI).

**Methods:**

A mixed-method design was used, consisting of a self-report questionnaire, numerous images taken by users, and a series of focus group discussions. The patients were involved in an individualized training programme.

**Results:**

Nine MCI patients and their caregiver relatives were included. They showed good acceptance of the camera and downloaded an appropriate number of images on a daily basis. Perceived severity and ease of use were the main factors associated with the intention to use the device.

**Conclusions:**

Older adults with MCI can become competent users of lifelogging wearable cameras with a good level of acceptance. Privacy concerns are outweighed by the potential benefits for memory. Limitations, strengths and implications for future research are discussed.

**Electronic supplementary material:**

The online version of this article (10.1186/s12877-019-1132-0) contains supplementary material, which is available to authorized users.

## Background

Mild Cognitive Impairment (MCI) refers to a condition affecting people who suffer memory impairment beyond what would be expected for their age, but who maintain relatively normal levels of general cognitive functions and are able to carry out activities associated with daily life. When those suffering this condition are followed through longitudinal studies, a significant proportion progress to clinically probable Alzheimer’s Disease, which is the most common form of dementia [1]. Numerous interventions have been proposed to help delay or reverse the progression to dementia in people with MCI but, to date, no pharmacological therapies have proven effective [2]. In recent years, several solutions incorporating Information and Communication Technology (ICT) have been developed with the aim of compensating for decreased cognitive abilities in people with dementia. Research has shown that environmental and assistive technology is capable of developing cognitive prostheses specifically aimed at promoting and supporting the autonomy, well-being and functionality of people with dementia [3, 4].

A new Assistive Technology Device (ATD), in the form of a lifelogging camera, has been developed for use as a memory prosthesis. This is a small camera, worn around the neck, which automatically takes hundreds of pictures per day from a first-person perspective. These images can then be easily uploaded onto a computer and viewed in relatively quick succession, providing a rich set of photos of an individual’s daily activities [5, 6]. Recent research has shown that reviewing daily images improves the recall of episodic memory in clinically impaired populations, such as people suffering from anterograde amnesia due to brain injury, alcohol-related brain damage, or Alzheimer’s Disease [6–9]. The therapeutic potential of using a lifelogging camera as a therapeutic intervention to improve memory has gained interest in recent years, although research in this area has so far been limited by small sample sizes and single-case research designs [10, 11]. Very few studies have hitherto explored the benefits of using lifelogging cameras in patients diagnosed with MCI or Alzheimer’s disease, although promising results have been reported, with identified improvements in recall and confidence and a reduction in anxiety [8, 12, 13].

In recent years, the public health sector has shown a growing interest in the use of lifelogging wearable cameras as an aid for memory, but new dilemmas have emerged relating to privacy and usability. This approach is of notable importance since such cameras allow patients to automatically capture continuous images while they are carrying out their daily activities [14, 15]. To date, little is known about the kinds of privacy challenges that these new devices are likely to pose, although several studies have highlighted factors that influence the use of such technology. Negative perceptions of their ease of use and utility are the main obstacles that seem to be involved, judging from questions concerning user acceptance of this technology [16]. Several studies have also sought to investigate the acceptance of such technology by patients, and particularly older people, as it has been reported that they may be reluctant to use therapeutic tools incorporating new technology [17]. Similarly, previous research has shown that people with MCI have a reduced ability to manage everyday technology and often perceive it as significantly more difficult to use than those without cognitive impairment [18]. In this sense, designing ATDs for people with MCI requires consideration of their capabilities and limitations, including their need for, and acceptance of, technological support. These factors may influence the engagement of such therapies and also the results of programmes including their use [19]. Despite evidence of this need, there has previously been a notable lack of research in this area, and particularly related to the use of ATDs by the population suffering cognitive impairment [4].

This research was designed as an exploratory study to investigate the use and acceptance of an approach involving the use of a wearable lifelogging camera by older adults with MCI. It was organized in order to remedy the previous dearth of information and prior knowledge relating to this topic. This constitutes a previous, but necessary, stage within the research conducted by our group (“ReMemory, a cognitive training tool based on life-logging for mild cognitive impairment” -http://www.rememory.cat-). The next phase of the research will focus on checking the effectiveness of a cognitive intervention programme for people with MCI based on reviewing images of daily episodes captured by a lifelogging wearable camera. The images captured by those wearing the cameras will be then used to stimulate episodic memory as part of a cognitive intervention programme.

In the present study, which is the initial phase of the ReMemory project, we were particularly interested in discerning whether older adults with MCI could competently use the lifelogging wearable camera and in knowing their intention to use it. We also wanted to explore other factors associated with the use of wearable cameras, such as checking whether privacy concerns could constitute a significant limitation to their use. Our working hypothesis was that, with appropriate training and information, older adults would be both able and willing to use wearable cameras. We also anticipated that privacy concerns could be relevant and could reduce the willingness of some patients to use them.

To better understand this scarcely studied issue, we used a mixed-method research design, integrating both quantitative and qualitative data. It was thought that the quantitative data would provide comparable imput, while the qualitative data would provide a better explanation of the meaning behind the numbers.

## Methods

### Design

This was a mixed-method study that involved the use of both quantitative and qualitative components. The quantitative measures included a self-report questionnaire on the acceptance of technology and the computation of useful images recorded and downloaded by each subject on a daily basis. The qualitative input consisted of analysing focus group discussions centred on issues related to the acceptance of cameras and privacy. The results of both the quantitative and qualitative analyses were jointly interpreted, as complementary approaches, in pursuit of the study objectives.

### Materials

The lifelogging camera used in this study was a Narrative Clip® (http://getnarrative.com): a small, wearable camera which automatically takes pictures every 30 s. It is small (36x36x9 mm or 1.42 × 1.42 × 0.35 in.) and light (weighing about 20 grammes or 0.7 oz). The camera is hung around the patient’s neck, using a necklace or piece of string, and takes images of everything that they do throughout the day. At the end of each day, the patient – or a helper - must then download the images, at home, by simply connecting the camera to a Universal Serial Bus (USB) port on a computer. They must also leave the camera connected to their computer to recharge the battery.

### Subjects

The study included 9 patients who had been diagnosed with MCI and their informal caregivers. The patients were recruited from two adult day centres for people with MCI and mild dementia located in Terrassa (Spain). They were asked to collaborate in this study in order to test a wearable camera and help ascertain whether it could be appropriately used by people with MCI to obtain personal images of day-to-day events that could subsequently be used as part of a treatment to help improve their memory. The inclusion criteria were: a) age 65–90; b) diagnosis of MCI by a neurologist; c) meeting the criteria for single domain amnestic MCI [1, 20]; d) having a caregiver or reliable informant who could monitor the patient’s daily activities; and e) having received cognitive training for three months or more at an adult day centre. The exclusion criteria were: a) an unstable medical condition; b) a presence, or history, of severe psychiatric disorder, cerebrovascular disease or neurological disorder; c) illicit drug or alcohol abuse or dependence; d) relevant hearing, vision, motor and/or language deficits; e) fewer than 4 years of formal education; and f) an insufficient knowledge of the Spanish language. The criteria chosen included the homogeneity of the functional and clinical characteristics of the sample and an appropriate level of heterogeneity relating to gender, level of education and knowledge of technology.

### Procedure

The study was conducted in four phases.

**Fig. 1:**
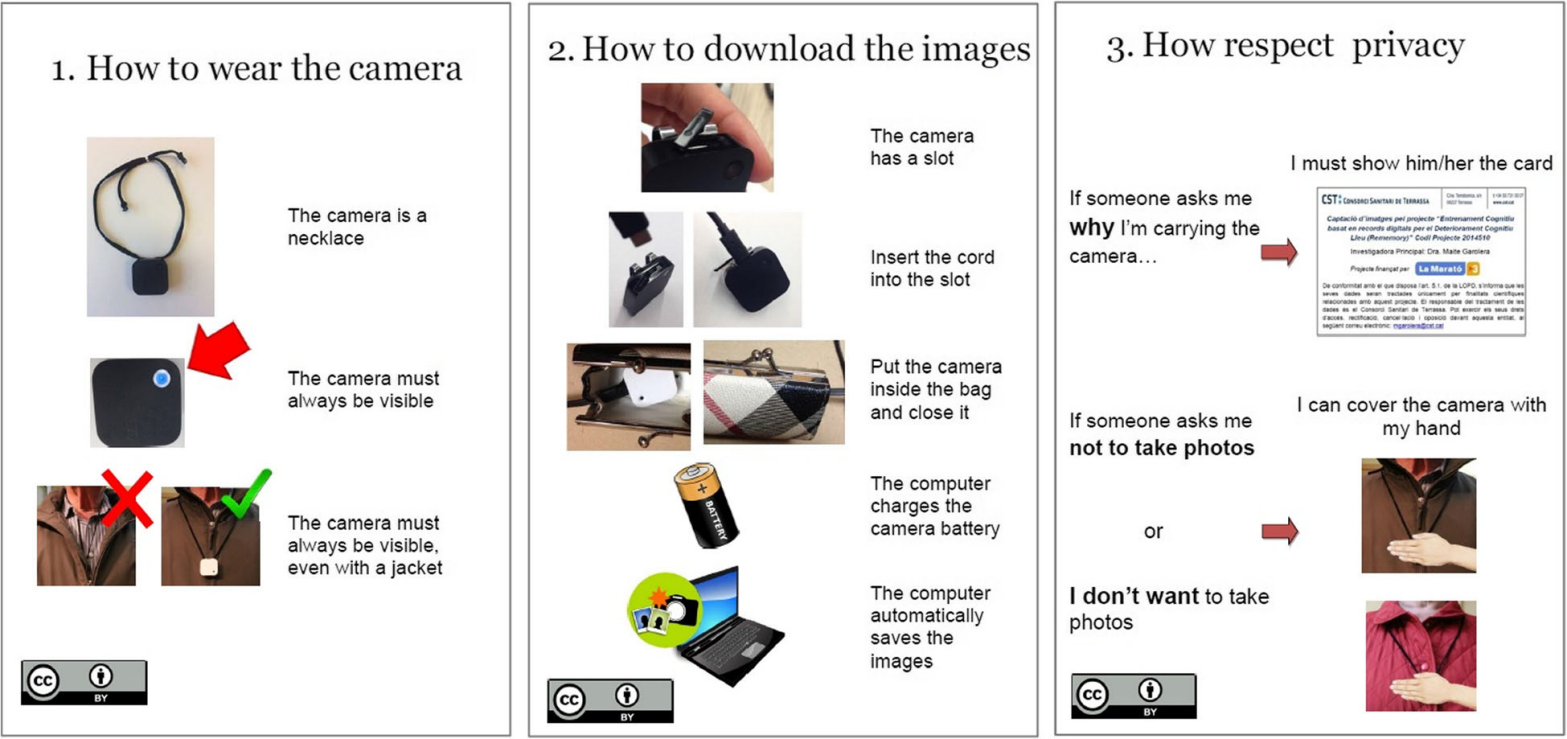
Training instruction sheets given to the patients, including instructions about the whole intervention programme procedure. Note: the images were created by the authors, except for the images of the battery, camera and laptop (sheet number 2), which were extracted from a website that provides a database of free pictures (www.pd4pic.com)

Phase I consisted of adapting the training material to meet Patient Education Materials Assessment Tool (PEMAT) guidelines [21]. The training material used in the study included several information sheets containing instructions on all the procedures that the patients needed to perform (Fig. 1). The material was reviewed by 10 subject experts (three patient caregivers, two neuropsychologists, two occupational therapists and two experts in Human-Computer Interaction (HCI) who completed the PEMAT inventory. This material was later given to the patients for reference.

Phase II consisted of providing training. The patients all received one hour of individualized training on how to use the camera and upload the images; they were also given abbreviated instructions to take home and uses as a reference whenever needed. The patients were also advised not to wear the cameras in public toilets, changing rooms or banks and told that they could opt not to take photos by simply covering the camera lens.

Phase III involved the patients using the camera in real-life situations. This phase lasted seven days. During this time, they had to wear the camera throughout the day and connect it to a computer to charge the battery and upload images every night.

Phase IV consisted of collecting and analysing quantitative and qualitative information relating to the experience.

### Data collection

Sociodemographic data about the patients and caregivers and data relating to patients’ cognitive impairment, depression symptoms and experience in the use of technology were collected at the baseline. The Mini Mental State Exam (MMSE) was used to evaluate the level of patients’ cognitive impairment [22] and the Geriatric Depression Scale was used to quantify the intensity of any symptoms of depression [23]. Following Rubin & Chisnell [24], the patients were categorized as having low, medium or high levels of experience in the use of technology. This was done through specific questions that were presented to them and related to the previous use of similar appliances.

Patient acceptance of technology was assessed using the integrative framework from the Wearable Technology Acceptance in HealthCare (WTAH) survey developed by Gao [25]. This is an instrument designed to evaluate the factors associated with a user’s willingness to adopt wearable technology in healthcare. It is based on the unified theory of acceptance and the use of technology 2 (UTAUT2) [26], the protection motivation theory (PMT) [27], and the privacy calculus model [28]. In this research, we slightly adapted Gao’s original construct in order to fit it into the context of using a wearable lifelogging camera to improve memory. Each construct contained 3 items and each item was measured on a five-point Likert scale, with scores ranging from 1 (“strongly disagree”) to 5 (“strongly agree”). The last construct used, called the Behavioural Intention (BI), was the dependent variable. The different constructs are described in Table 1. A composite score was obtained for the different constructs by calculating average scores for the 3 items used in each construct. To ensure that the patients had correctly understood the content of the survey items, a member of the research team was present when each patient completed the survey and helped them to resolve any questions and to clarify the content. The WTAH survey has been made available as Additional file 1.

**Table 1:** Constructs included in the Wearable Technology Acceptance in Health Care (WTAH) survey

*Performance Expectancy (PE)*	The degree to which using this technology will bring effectiveness to users in performing daily activities
*Hedonic Motivation (HM)*	The pleasure or enjoyment derived from adopting and using the technology
*Effort Expectancy (EE)*	The degree of ease related to the patient’s use of the technology
*Functional Congruence (FC)*	Product quality in terms of comfort, fashion and reasonable pricing
*Self-Efficacy (SE)*	The patient’s capacity to use the wearable device to self-monitor and self-manage their memory functioning
*Social Influence (SI)*	The extent to which the patient’s decision-making is influenced by others’ perceptions
*Perceived Vulnerability (PV)*	The possibility of the patient experiencing memory problems
*Perceived Severity (PS)*	The extent of the threat from behaviour that is unhealthy for memory and the importance of following medical prescriptions that are good for memory
*Perceived Privacy Risk (PPR)*	The patient’s perceptions of risk when professionals attempt to collect, use, and distribute information about them and their behaviour
*Behavioural Intention (BI)*	The intention to use the device in the future

Adapted from Gao [22]

Another quantitative analysis consisted of computing the rate of obtaining useful images with respect to the total number of images recorded and downloaded by each subject on a daily basis. This measurement was used to assess the extent to which the patients had used the camera correctly and to check whether an adequate number of images had been obtained to represent significant daily episodes that could subsequently be used in the cognitive intervention programme. The task of selecting the images was performed by computer vision tools that contained a deep learning algorithm. This enabled them to organize the large-scale collection of images captured by the patients on a daily basis [29]. The deep learning algorithm was specially designed to automatically discriminate between informative and non-informative images. It was also programmed to discard non-informative images, such as dark images, blurred images or images without content, such as when the camera photographed a wall, the ground, a ceiling, or the sky, etc., and also any repeated images. The cognitive intervention of the ReMemory Project requires 60 informative images for each daily episode. Assuming that the camera will collect a large number of non-informative images (we calculate that approximately 75% of the images captured tend to be non-informative given that the camera automatically takes images once every 30 s), it is expected that each patient should be able capture at least 300 relevant images per day.

The qualitative data were obtained from two focus groups (one for each patient centre) in which the patients and their caregiving relatives participated. The duration of each focus group’s activity was approximately 90 min and both focus groups were led by the same person. This person, who was a member of the research team and had relevant experience in organizing this type of task, acted as moderator for these groups. The group leader followed a pre-established script and facilitated discussion. They also had the support of an assistant moderator, who took notes throughout each session. The resulting script was structured around three major topics: a) ease of use of the camera; b) privacy, and c) a general assessment: acceptance, expectations and recommendations for improvement. The proceedings of the focus groups were recorded and transcribed.

### Quantitative and qualitative analysis

Means and standard deviations were used to describe the quantitative variables, while absolute frequencies and percentages were used for qualitative measurements. In addition, and in light of the relatively small size of our sample, we performed exploratory correlation analyses in order to examine any potential association between the Behavioural Intention construct of the WTAH and the rest of the constructs, as well as investigating the sociodemographic and clinical variables of the patients studied. This was done using Spearman’s nonparametric correlation.

A thematic qualitative analysis was carried by the same person who led the focus groups and was performed from a socio-constructivist perspective [30]. It focused on understanding how patients constructed and interpreted the experience of bringing the camera into their daily lives. The analytical procedure was an adaptation of the steps suggested by Braun and Clarke [31]: a) reading and re-reading for familiarization with the data and the generation of initial codes; b) discussion and initial re-coding of the codes; c) code analysis and grouping data into categories based on thematic criteria; and d) discussion of the categories and (e) report writing.

## Results

### Patient characteristics

The characteristics of the patients are presented in Table 2.

**Table 2:** Descriptive characteristics of the sample (percentages or means and SDs)

	Patients (*n* = 9)	Caregivers (*n* = 9)
Age	70.3 (5.38)	68.3 (5.74)
Sex (%)		
male	56.0	44.0
female	44.0	56.0
Education (%)		
high (university or college)	22.0	0.0
medium (secondary school)	11.0	56.0
low (primary or lower)	67.0	44.0
Marital Status (%)		
married	89.0	89.0
widow/er	11.0	11.0
Relationship patient-caregiver (%)		
parents & adult children	11.0	
spouses	89.0	
Living conditions (%)		
living alone	11.0	
living with spouse	78.0	
living with spouse and children	11.0	
MMSE (mean scores)	26.1 (2.15)	
Yesavage Depression Scale (mean scores)	11.4 (5.92)	
Knowledge of technology (%)		
high	22.0	
medium	56.0	
low	22.0	

### Wearable technology acceptance in HealthCare survey (WTAH)

**Fig. 2:**
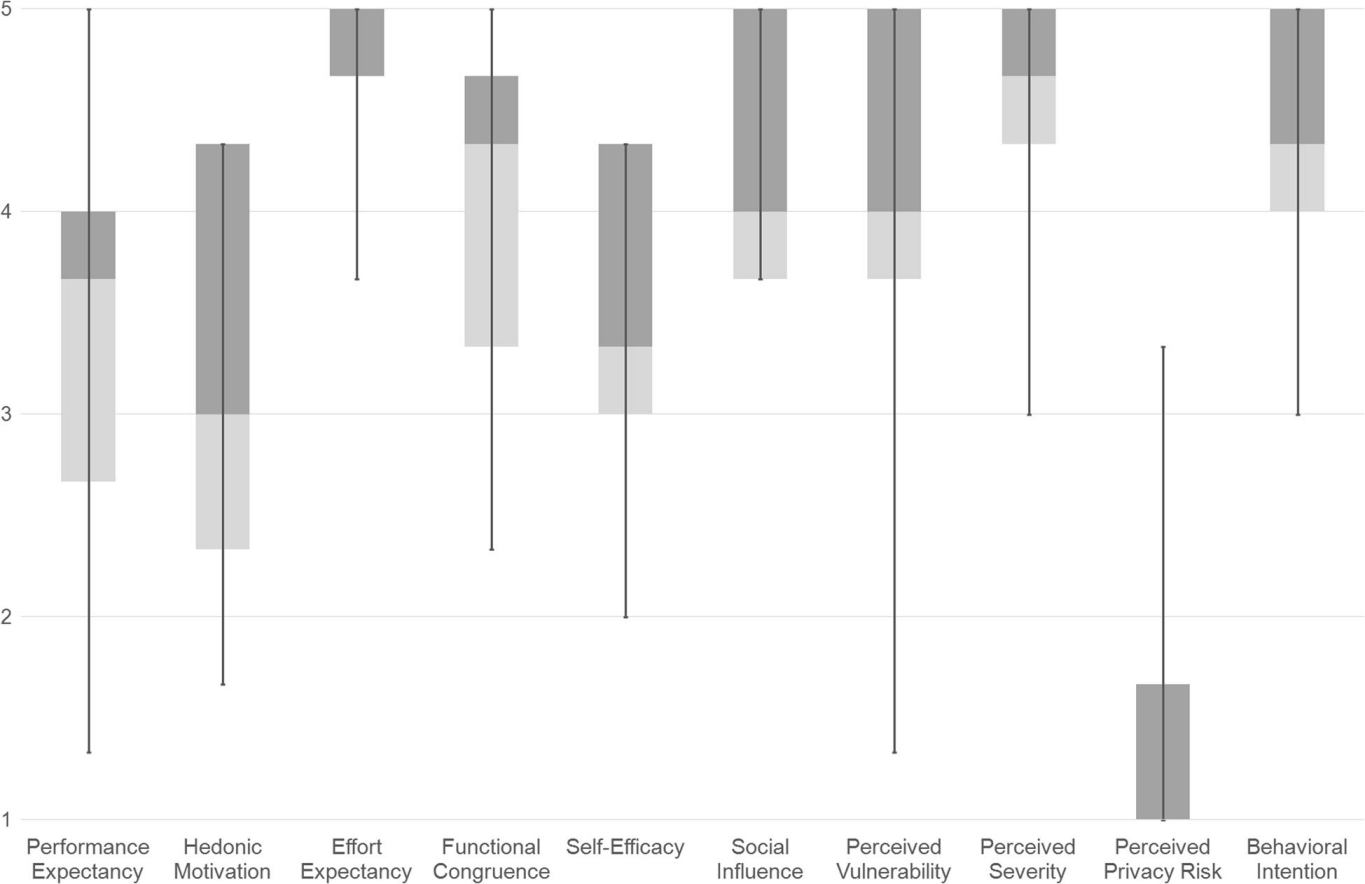
Boxplot showing the distribution of the WTAH constructs for the sample. Note: The bottom and top vertical lines show the lower and upper scores. The top of the rectangle (dark grey colour) corresponds to the third quartile; the horizontal line near the middle of the rectangle indicates the median; the bottom of the rectangle (light grey colour) relates to the first quartile

The results of the WTAH survey showed that the patients exhibited a good level of acceptance of the camera; a mean score of 4.2 (SD 0.8) was recorded in the Behavioural Intention construct. Moreover, the patients considered the camera easy to use; a mean score of 4.7 (SD 0.4) was recorded in the Effort Expectancy construct. The results also showed a low perceived privacy risk in the exchange of information about memory difficulties; a mean score of 1.5 (SD 0.9) was recorded in the Perceived Privacy Risk construct. The distribution of the scores for each construct is shown in Fig. 2.

In terms of associations between sociodemographic and clinical factors and the BI construct, we only identified a strong association with the Geriatric Depression Scale (*r* = 0.93, *p <* 0.01). The factors measured by the WTAH survey that showed associations with the BI construct were Perceived Severity (*r* = 0.87, *p <* 0.01) and Self-Efficacy (*r* = − 0.70, *p <* 0.05). No significant relationships were found between the BI construct and the remaining constructs of the WTAH. We also found a significant association between Geriatric Depressive Symptoms and the Perceived Severity construct (*r* = 0.92, *p <* 0.01).

### Computation of the images downloaded

On average, each patient recorded 935 images a day and the algorithm discarded 46% of the total number of images downloaded by each patient. The total number of images obtained by each patient was considered appropriate since it exceeded the pre-established minimum of 300 images per day. Due to technical problems involving filing on the first day of the procedure, 1 day of recordings was lost for 4 of the users (see Table 3).

**Table 3:** Results of the images downloaded each day and kept by each patient

Patient	Valid days	Total images downloaded per day	Images kept (%)
1	7	1115	743 (67%)
2	6	960	668 (70%)
3	7	994	546 (55%)
4	6	885	309 (35%)
5	6	1411	644 (46%)
6	6	693	398 (57%)
7	7	838	495 (59%)
8	7	702	390 (56%)
9	7	820	363 (44%)
Mean for the total sample	6,5	935,3	506,2 (54,3%)

### Focus group

Two focus groups were organized with the participants. The results were classified into the following three categories:

- Learning to wear the camera. Most of the participants (both caregivers and older adults with MCI) considered that it was easy to use the camera during the test. However, the participants also referred to the fact that using the camera entailed a learning process during which they needed to be trained and they highlighted certain factors that facilitated the learning process. First, the previous training session helped them to become familiar with the technology. Second, they said that consultations with a professional during the study enabled them to allay doubts and to overcome problems, most of which related to the use of the technology. Finally, in some cases, the MCI patients said that the support and supervision provided by their caregiver in certain tasks or actions, such as connecting and loading the camera, were also key for them continuing with the experiment (see Table 4).

**Table 4:** Sample of responses organized by theme and sub-theme

Topics	Subtopics	Illustrative Quotations
LEARN HOW TO WEAR THE CAMERA	*Learning*	Patient 04: *Well, the first time, the first time I tried to open the clasp on the cord, I didn’t turn it as I should have and I wasn’t able to open it…but from then on…* Caregiver 04: *We told him to look at it as if it was a ring and from then on, everything went very well (US04, FUS04, focus group 1).*
		Caregiver 09*: on the first day, we couldn’t connect the cable to the USB port, we tried but it didn’t fit. Then we tried the middle port and it fitted perfectly (US09, FUS09, focus group 2).*
	*Training session*	Caregiver 02*: During the session the trainer explained everything well and therefore everything went well (FUS02, focus group 1).*
	*Support of caregivers*	Patient 03*: When I couldn’t do something, for whatever reason, he did it and that was it! (US03, focus group 1).*
RELUCTANCE TO USE THE CAMERA	*It can be seen*	Patient 03*: Yes, people look at you* Patient 04*: I sometimes had to cover the camera, ... but* Patient 01*: When you interact with other people, if people are close to you, they understand it well. But if people don’t know you, they look at you as if they are thinking “well, what is he doing?”, it’s a study...in the end, when I found myself in that situation, I took the camera off and I put it in my pocket because I thought that people were uncomfortable with all of this (US01, US03, US04, focus group 1).*
	*It’s watching us*	Caregiver *01: It was like the reality show “Big Brother” [...] the camera watches everything, including relatives, or in the kitchen, if someone is cooking, or if there are things to wash, when you open the cupboards ...* Patient 02*: Yes, yes, it makes you feel a bit uneasy, everybody can see everything (FUS01, US02, focus group 1)*
	*Emotional impact*	Patient 01*: The truth is that I wasn’t bothered. Well you’re always a little aware of the camera, but when I took it off, I felt relieved... (US01, focus group 1).*
ACCEPTANCE OF THE CAMERA	*Decisional balance*	*Patient 02: I believe that this camera will serve us or those who come after us. To not lose our head even more* *Patient 01: I think it’s positive for us, because it can correct us when we fail and help us to improve* *Patient 05: Although my family and I were sometimes bothered by the camera, there were moments that I didn’t remember having it on me. In fact, I’ve gone about my normal life without thinking about the camera.*
	*Recommendations*	*Patient 03: the camera is black and people look at it a lot. I think that if it were a more natural colour, people would look at it less*.
- Reluctance to use the camera. Some of the patients with MCI said that they felt embarrassed or were worried about the comments that the camera might provoke, or that they hid the camera depending on where they went. The camera can also capture certain intimate aspects of day-to-day life (e.g. personal hygiene, eating habits, going to the toilet) that could be a threat to privacy. Being aware that they could be recorded in their intimacy causes people feelings of vulnerability and arouses in then negative emotions that may make them reluctant to wear the camera. However, despite this situation, none of the patients changed their daily activities and the majority even forgot that they were wearing the camera. Whatever the case, most of the patients and their caregivers reported that they felt relieved when the study ended, which supports the idea that there is an emotional load associated with wearing the camera (see Table 3).
- Evaluating the acceptability of the camera. The vast majority of the patients with MCI regarded recording their daily life as useful, as this helped them to receive more personalized memory treatment. Furthermore, they were able to compare the potential difficulties, or threats, associated with wearing the camera with the advantages. Most of the participants stated that despite the inconveniences encountered, the therapeutic benefits, ease of use, and autonomy of being able to turn the camera off in situations of privacy or discomfort, provided sufficient reasons for acceptance. The majority of the recommendations made by the participants (whether family carers or older adults with MCI) related to reducing the difficulties in its use. These included questions such as improving the way in which the camera is worn, or the design of its string, incorporating a signal to differentiate between the front and the back of the camera, and organizing a tutorial on how to use the camera (see Table 3).

## Discussion

This is a piece of preliminary research which constitutes the first stage of a larger project that aims to develop a cognitive intervention programme for people with MCI based on images captured by a lifelogging wearable camera. The main purpose of the current stage of the project was to contribute to the existing body of knowledge about patient acceptance of using the wearable lifelogging camera in older people with MCI. This was designed as an exploratory study to help define the problem more precisely, given the previous lack of adequate knowledge about this subject. The convergent design that summarizes the quantitative and qualitative results provided a more solid basis for data collection and helped to improve the explanation of some of the numerical results and to compare some of the qualitative perceptions, in the same way in which they have previously been reported in the literature [32].

The first conclusion from the study allows us to answer our first question about the ability of older adults with MCI to use this technology efficiently. We have demonstrated that they can be competent users of a lifelogging wearable camera designed to record their everyday activities. Our analysis of the computed rate of useful images downloaded by each subject on a daily basis showed us that they were all able to record and download images from day to day and that the set of images obtained for each subject was sufficient to obtain a significant set of images of their daily life. The comments registered during the focus groups further confirmed this appreciation. This conclusion minimizes some of the technological difficulties that some other authors had predicted that people with MCI would report when using this technology [18]. It is important to underline that the present study involved a well-structured training programme, appropriately adapted training material, and continued support provided by professionals. The relevance of these factors was also highlighted by most of those participating in the focus group discussions. We are convinced that the approach was capable of minimizing the disabling consequences of the conditions affecting the patients and that this favoured the success of the experience.

Regarding the willingness of patients to use the device, the results were partly in line with the PMT and UTAUT2 models. PMT is a social cognitive model that argues that the willingness to adopt practices associated with healthy behaviour will be determined by the severity of the illness, perceived vulnerability, and an appraisal of the efficacy of certain practices constituting healthy behaviour [27]. UTAUT2 is a model that explains behaviour associated with the use of health-related technologies. It defines the following constructs that are implicit in behaviour: expectations concerning performance, expectations relating to effort, social influence, hedonic motivation, cost effectiveness and habit [26, 33]. The combined application of quantitative and qualitative methods used in our study revealed that the perceived severity of the process and expectations relating to the effort required (or ease of use, as explored in the focus groups) were the main factors that could explain the willingness of the patients consulted in our sample to use the device. The association between the intention to use the device and severity of depressive symptoms could be explained by the high correlation between the latter and Perceived Severity. Contrary to previous findings, the quantitative results of this study showed a negative association between self-efficacy and behavioural intention. Even so, the qualitative analysis allowed us to interpret this apparently paradoxical result. We found that some patients were more reluctant to use the wearable camera than others despite having a high level of self-efficacy. When we checked, we found that these people led very active social lives and we think that this could explain their reduced willingness to use the device. Due to the relatively small sample size and the exploratory nature of the statistical analyses undertaken, these results must - at least for the moment – be regarded as merely preliminary. Nevertheless, our findings provide initial arguments to support the case for recognizing the perception of a problem and show the ease of use as a potential solution; these are relevant factors for understanding the willingness of patients to adopt this procedure.

The qualitative analysis performed in this study provided us with sufficient information to conclude that concern over privacy is an important factor and one that must be considered. The patients expressed certain feelings of discomfort and vulnerability relating to the automatic capture of all aspects of their life experience and they thought that this implied an invasion of their privacy. Some of them also stated that they felt a sensation of relief when the study ended. Previous studies had also reported the reluctance of patients to use this type of device because of privacy concerns [34] and the refusal of subjects to wear the camera for long periods [35]. Whatever the case, the potential benefits for memory which are expected to accrue from using the device and the ability to decide when and when not to take pictures helped the patients in our sample to overcome their initial reluctance to use the device based on invasion of privacy. Similar results were reported by Mattheus et al. [36] in their study of the usability of a wearable camera system for family caregivers working with patients suffering from dementia. We believe that acceptance of the technology used in this study: the Unified Theory of Acceptance Use of Technology (UTAUT) [26], should be complemented with other measures to fully allay any potential user and third-party concerns.

### Limitations of the study

Some limitations must be considered when interpreting our data. The first, and perhaps most important, was the small sample size; it was reduced at the expense of incorporating a demanding methodological design. This research should, however, be considered as what it was: an initial, exploratory study. Our data should therefore be regarded as a series of preliminary observations that will need to be confirmed in larger cohorts of MCI patients. Despite its small size, the variables used to describe the sample maintained their homogeneity and discursive heterogeneity with respect to the objectives of the study, in terms of age, gender, educational levels and different levels of knowledge of the technology employed. A range of views were obtained, and data saturation was achieved after the second focus group. Another limitation that must be considered was the short duration of the experience: the trial only lasted for 7 days. Further research over a longer period will be needed to validate these results. Finally, the lack of the measures on the quality of the images as well as the value to the participants from reviewing the images are other limitations of these research that must be taken into account.

### Strengths of the study

On the positive side, it is possible to highlight several strengths of this study. Firstly, it is innovative, as there has hitherto been an absolute lack of standardized research into patient acceptance of lifelogging cameras. Secondly, the methodological quality of this study was rigorous. Thirdly, the results obtained have significant clinical relevance. We have shown that the wearable lifelogging camera can be a useful device which is easy to use for older people with MCI. This is something that had not been adequately demonstrated previously. This is an important question because it helps to lay down the foundations for our next study which will allow us to assess whether the images captured by the camera can be used in therapy within programmes for stimulating episodic memory.

Overall, the complementarity of the data gathered helped to detect some elements that may otherwise have hindered the use of this technology. This also helped to improve the protocol and to test some of the instruments used to gather data about acceptance. In particular, we learnt the importance of reviewing quantitative results in conjunction with qualitative explanations, as well as the need to delve into private concerns and to find out more about how the patients valued the training sessions. All of this will be taken into account and improved upon in future stages of the project. Future studies will similarly contemplate the possibility of patients being able to select their own images in conjunction with an algorithm. This would allow them to keep what they consider to be the most significant and meaningful images. Furthermore, real-life implementation of wearable lifelogging systems should consider their use over relatively short periods since fatigue and overload seemed to be important issues and ones that could otherwise limit their use. In future research, it will be necessary to explore all of these questions in greater depth, and especially those regarding privacy; this could even extend to bystanders. It would also be advisable to expand the study of the acceptability of this device to other people with cognitive impairment, such as patients with dementia or brain injuries.

## Conclusions

These results demonstrate that lifelogging wearable cameras can be used by older people with MCI as long as they are accompanied by a well-structured programme including specially adapted material. The recognition of the existence of a problem and the perception that the device can be of help were key elements in favour of it, as was its ease of use. This led us to the conclusion that the wearable lifelogging camera is an interesting device whose potential utility as a therapeutic aid to memory for older people with MCI merits further investigation.

## Additional file

Additional file 1: Wearable Technology Acceptance in Health Care Survey. Based on Yiwen Gao, He Li & Yan Luo, 2015. This is the Wearable Technology Acceptance in Health Care Survey (Gao et al., 2015), adapted by the authors to fit it into the context of using a wearable lifelogging camera to improve memory. (DOCX 33 kb)

## Abbreviations

ATD: Assistive technology device
BI: Behavioural intention
EE: Effort expectancy
FC: Functional congruence
HCI: Human-computer interaction
HM: Hedonic motivation
ICT: Information and communication technology
MCI: Mild cognitive impairment
MMSE: Mini mental state exam
PE: Performance expectancy
PEMAT: Patient education materials assessment tool
PMT: Protection notivation theory
PPR: Perceived privacy risk
PS: Perceived severity
PV: Perceived vulnerability
SE: Self-efficacy
SI: Social influence
USB: Universal serial bus
UTAUT: Unified theory of acceptance and use of technology
WTAH: Wearable technology acceptance in healthCare
