# Supplementary material for: Acceptability of a lifelogging wearable camera in older adults with mild cognitive impairment: a mixed-method study
Source: BMC Geriatr. 2019 Apr 16;19:110. doi: 10.1186/s12877-019-1132-0 (PMC6469032; doi:10.1186/s12877-019-1132-0)
Supplement: Supplementary file 1 — Wearable Technology Acceptance in Health Care Survey. Based on Yiwen Gao, He Li & Yan Luo, 2015. This is the Wearable Technology Acceptance in Health Care Survey (Gao et al., 2015), adapted by the authors to fit it into the context of using a wearable lifelogging camera to improve memory. (DOCX 33 kb) [file 12877_2019_1132_MOESM1_ESM.docx]

**Wearable Technology Acceptance in Health Care Survey**

Based on  Yiwen Gao, He Li & Yan Luo, 2015

*DATE:*

***INSTRUCTIONS****:*

*Rate each option from 1 (strongly disagree) to 5 (totally agreement).*

|  | Strongly Disagree | Disagree | Do not agree / disagree | Agree | Totally Agree |
| --- | --- | --- | --- | --- | --- |
| 1. **Performance expectancy (PE)** | 1 | 2 | 3 | 4 | 5 |
| *PE1*: I find the wearable camera useful to remember things in my daily life. |  |  |  |  |  |
| *PE2:* using the wearable camera useful helps to remember things more quickly. |  |  |  |  |  |
| *PE3:* using the wearable camera improves the quality of my daily healthcare seeking. |  |  |  |  |  |
|  |  |  |  |  |  |
| 1. **Hedonic motivation (HM)** | 1 | 2 | 3 | 4 | 5 |
| *HM1*: using wearable camera is fun. |  |  |  |  |  |
| *HM2:* using wearable camera is enjoyable. |  |  |  |  |  |
| *HM3:* using wearable camera is entertaining. |  |  |  |  |  |
|  |  |  |  |  |  |
| 1. **Effort expectancy (EE)** | 1 | 2 | 3 | 4 | 5 |
| *EE1:* learning how to use wearable camera is easy for me. |  |  |  |  |  |
| *EE2*: I find wearable camera easy to use. |  |  |  |  |  |
| *EE3:* It is easy for me to become skillful at using wearable camera. |  |  |  |  |  |
|  |  |  |  |  |  |
| 1. **Social influence (SI)** | 1 | 2 | 3 | 4 | 5 |
| *SI1*: people who are important to me would think that I should use the wearable camera |  |  |  |  |  |
| *SI2*: people who influence me would think that I should use the wearable camera |  |  |  |  |  |
| *SI3*: people whose opinions are valued to me would prefer that I should use the wearable camera |  |  |  |  |  |
|  |  |  |  |  |  |
| 1. **Functional congruence (FC)** | 1 | 2 | 3 | 4 | 5 |
| *FC1:* wearable camera is expected to be comfortable. |  |  |  |  |  |
| *FC2*: wearable camera is expected to be fashionable. |  |  |  |  |  |
| *FC3:* wearable camera is expected to be priced appropriately considering their quality. |  |  |  |  |  |

|  | Strongly Disagree | Disagree | Do not agree / disagree | Agree | Totally Agree |
| --- | --- | --- | --- | --- | --- |
| 1. **Self-efficacy (SE)** | 1 | 2 | 3 | 4 | 5 |
| *SE*1: it is easy for me to self-monitor my memory performance by using wearable camera. |  |  |  |  |  |
| *SE2*: I have the capability to use wearable camera to self-monitor my memory performance |  |  |  |  |  |
| *SE3:* I am able to use wearable camera to self-monitor my memory performance without much effort. |  |  |  |  |  |
|  |  |  |  |  |  |
| 1. **Perceived vulnerability (PV)** | 1 | 2 | 3 | 4 | 5 |
| *PV1:* I am at risk for suffering memory problems. |  |  |  |  |  |
| *PV2*: it is likely that I will suffer memory problems. |  |  |  |  |  |
| *PV3:* it is possible for me to suffer memory problems. |  |  |  |  |  |
|  |  |  |  |  |  |
| 1. **Perceived severity (PS)** | 1 | 2 | 3 | 4 | 5 |
| *PS1*: if I suffered memory problems, it would be severe. |  |  |  |  |  |
| *PS2*: if I suffered memory problems, it would be serious. |  |  |  |  |  |
| *PS3:* if I suffered memory problems, it would be significant |  |  |  |  |  |
|  |  |  |  |  |  |
| 1. **Perceived privacy risk (PPR)** | 1 | 2 | 3 | 4 | 5 |
| *PPR1:* it would be risky to disclose information about my memory performance to the people who provide me the wearable camera. |  |  |  |  |  |
| *PPR2*: there would be high potential for loss associated with disclosing information about my memory performance to the people who provide me the wearable camera. |  |  |  |  |  |
| *PPR3*: there would be too much uncertainty associated with giving information about my memory performance to the people who provide me the wearable camera |  |  |  |  |  |
|  |  |  |  |  |  |
| 1. **Behavioral intention (BI)** | 1 | 2 | 3 | 4 | 5 |
| *BI1*: I intend to use wearable camera device in the future. |  |  |  |  |  |
| *BI2:* I intend to use wearable camera device at every opportunity in the future. |  |  |  |  |  |
| *BI3:* I plan to increase my use of wearable camera device in the future |  |  |  |  |  |
